# Atypical Kawasaki Disease in a 16-Month-Old Baby: A Case Report and Literature Review

**DOI:** 10.7759/cureus.39336

**Published:** 2023-05-22

**Authors:** Sonali Singh, Pugazhendi Inban, Anshika Mishra, Anupam S Yadav, Tanveer Singh, Ramandeep Singh, Bansi Piyushkumar Savaliya, Saptak P Mankad, Chengala Ananyaa Gowthavaram, Aadil Khan

**Affiliations:** 1 Pediatrics, King George's Medical University, Lucknow, IND; 2 General Medicine, Government Medical College Omandurar, Chennai, IND; 3 Psychiatry, Ganesh Shankar Vidyarthi Memorial Medical College, Kanpur, IND; 4 College of Medicine, Sri Guru Ram Das University of Health Sciences, Punjab, IND; 5 College of Medicine, Punjab Institute of Medical Sciences, Punjab, IND; 6 College of Medicine, Crimean Federal University, Simferopol, RUS; 7 Internal Medicine, Shree Krishna Hospital, Anand, IND; 8 Internal Medicine, Malla Reddy Institute of Medical Sciences, Hyderabad, IND; 9 Internal Medicine, Lala Lajpat Rai Hospital, Kanpur, IND

**Keywords:** coronary aneurysms, transthoracic echocardiography (tte), polymorphous skin rash, luminal myofibroblastic proliferation (lmp), atypical kawasaki disease

## Abstract

Kawasaki illness is an inflammatory condition of small- to medium-sized vessels that primarily affects children. It affects the lymph nodes, skin, mucous membranes, and heart, especially the coronary arteries. Patients who lack the comprehensive clinical manifestations of classic Kawasaki disease (KD) are typically evaluated for incomplete KD. Such patients have persistent fever and lack one or more characteristic clinical signs. Here, we present a case of a 16-month-old baby presented with fever for nine days, excessive crying and irritability for four days, and refusal to feed for one day with pallor and developed lip cracking, mucositis, bilateral edema, and redness in the palms and soles followed by periungual desquamation. Lab evaluations revealed anemia, elevated white cell count, and c-reactive protein along sterile pyuria. Since the child became afebrile after ten days of illness, inflammatory marker levels decreased, and no coronary artery abnormalities were detected on 2D echocardiography, and the child was diagnosed with incomplete KD based on the clinical, laboratory, and radiological evaluations after ruling out all other possible causes. He was managed conservatively with low-dose aspirin, and the child was doing well on a two-month follow-up.

## Introduction

Kawasaki disease (KD) is an acute necrotizing vasculitides of medium-sized coronary arteries with a predisposition to coronary vessels. KD is currently the major cause of acquired heart disease among children in affluent nations, displacing acute rheumatic fever [1,2]. This disease is more common in children especially less than five years of age. Children of any race or ethnic group can get KD. It's more common in children whose families are from East Asia or Asian ancestry. It occurs in boys more often than in girls. KD is a widespread illness with varying incidence rates. Japan has the most significant incidence of KD, which has continuously climbed, with an annual rate of 308.0 per 100,000 children under the age of five reported in 2014 [3]. KD affects roughly 25 per 100,000 children under five in the United States [4]. A hospital in India reported an incidence rate of 48.4% for incomplete KD, which is similar to the rates published by most countries. The proportion of incomplete KD has been reported to be large in India which is around 41% [5].

The etiology of KD is still not established, and it is presumed to be related to waterborne or airborne bacterial and viral infections [6]. Specific genetic markers in children have also been linked to KD, such as human leukocyte antigen-B51 (HLA-B51) and HLA-Bw22j2 serotypes, gamma Fc region receptor III-A (FCGR3A) polymorphism of the immunoglobulin G (IgG), and haplotypes of chemokine receptor gene-cluster (CCR) 2-5. It also has a high incidence among siblings [7]. As per the immune response, the pathology has been linked to a response to a classic antigen that protects against future infections. Bronchial epithelial cells in children with KD isolate RNA form intracytoplasmic inclusions, which suggests a viral pathology [8]. The link between seasonal variation and higher incidence of the disease in the winter-spring season is pointed to evidence that the disease might be caused by the transportation of an agent inhaled by genetically predisposed children to the disease, triggering the immunological cascade [7]. The disease's predilection in five- to six-year-old youngsters may potentially impact younger or older age groups. Inflammation caused by KD is clinically significant in the coronary arteries, but it can potentially cause inflammation in the respiratory, gastrointestinal, urinary, dermatological, or nervous systems [9].

KD, a mucocutaneous lymph node syndrome, is an acute necrotizing vasculitis of medium-sized coronary arteries with a predisposition of coronary vessels. The immune response in KD is a cascade of systemic inflammation in multiple organs and systems affecting the medium-sized arteries during the acute febrile phase with clinical manifestations, which may include gastroenteritis, aseptic meningitis, interstitial pneumonitis, lymphadenopathy, pyuria, acute pancreatitis, and acute hepatitis [4,10]. Muscular arteries are typically affected by vasculopathy in medium-sized blood vessels. The following three interrelated processes define it: aneurysms result from necrotizing arteritis, which is the deterioration of the artery wall into the adventitia. Vasculitis lasts months to years and is characterized by lymphocyte, eosinophil, and plasma cell invasion but few macrophages. The myofibroblastic process can potentially produce stenosis, called luminal myofibroblastic proliferation. Moderately dilated and inflammatory arteries may recover after coronary artery damage. However, the pathological effects of coronary artery damage depend on the degree of the lesions produced in the vasculature. Giant saccular aneurysms without intima, medium, or elastica cannot regenerate. Rim rupture or subsequent thrombosis may cause the remnant adventitia to organize, recanalize, and calcify [2-6]. Diagnosis of KD is established clinically by clinical findings, and lab criteria may be used to supplement the diagnosis when it cannot be established clinically. To detect coronary aneurysms and other cardiac abnormalities, transthoracic echocardiography (TTE) is the imaging modality of choice.

## Case presentation

A 16-month-old baby was brought to the emergency department with chief complaints of fever for seven days, excessive crying and irritability for four days, and a refusal to feed for one day with pallor. He had a high-grade fever spike of 104'F and was intermittent with no aggravating and relieving factors. He was managed conservatively with intravenous fluids, and empirical antibiotics like vancomycin and gentamycin was also started after sending relevant cultures. After taking proper consent from the mother, the patient was examined in a well-lit room with appropriate exposure and following adequate hand hygiene. The child was conscious and irritable, with a heart rate of 140 beats per minute and a respiratory rate of 36 beats per minute. He was afebrile, all peripheral pulses were palpable, regularly regular, and normovolemic, and no radio-radial or radio-femoral delay was noted. A blood pressure of 94/62 mmHg was taken in the right upper. Mild conjunctival pallor was present, but icterus, cyanosis, clubbing, edema, and lymphadenopathy were absent.

All the necessary blood investigations revealed anemia for age, a high white blood count, raised inflammatory markers, and hypoalbuminemia (Table 1).

**Table 1:** The results of initial blood investigations

Parameter	Lab value	Reference range
White cell count	16500 /mm^3^	(4000-14000)
Hemoglobin	9 g/dl	(11-13.5)
Bilirubin	0.5 mg/dl	(0.1-1.2)
Serum albumin	2.5 g/l	(3.5-5.4)
Platelet count	170,000/mm^3^	(150,000-450,000)
Serum creatinine	0.3 mg/dl	(0.2-0.5)
Serum calcium	9.9 mg/dl	(9.0-10.6)
Blood urea nitrogen	14 mg/dl	(08-24)
Alanine aminotransferase	49 IU/L	(8-57)
Alkaline phosphatase	77 mg/dl	(36-95)
C-reactive protein	18 mg/l	(<10)
Erythrocyte sedimentation rate	25/hour	(3-13)
Lactate dehydrogenase	177 IU/L	(60-170)

**Figure 1:**
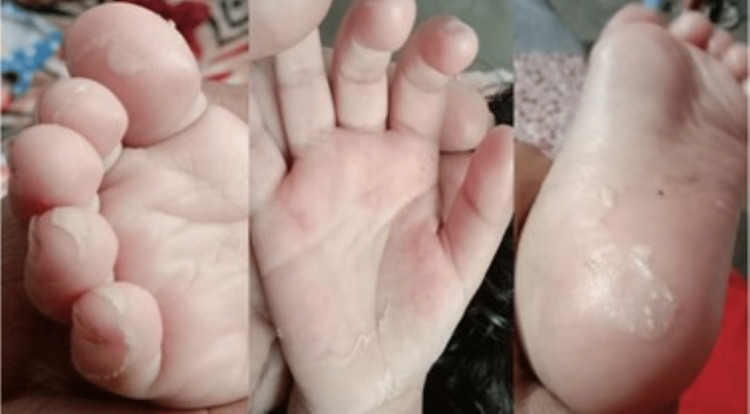
Periungual desquamation of the fingers and toes

Urine microscopy revealed sterile pyuria. Blood cultures and urine cultures were sterile. On day three of admission, the child developed edema over bilateral upper limbs, and edema of bilateral lower limbs increased. The mother also noticed redness over bilateral palms and soles, lip cracking, and red oral ulcers over the tongue and buccal mucosa. A dermatology consultation was made and asked to rule out KD disease as the child has some of the typical features of KD disease. Gradually, the fever subsided, and periungual desquamation of fingers and toes started on day nine of admission, as shown in Figure 1.

As there had been no episode of fever since the commencement of periungual desquamation and inflammatory markers were on a decreasing trend, the CRP started at 18 mg/l on the day of admission but increased significantly to 122.60 mg/l by the third day. However, after 10 days of treatment, the CRP had decreased to 22.18 mg/l, indicating improvement, and intravenous immunoglobulin (IVIG) was not transfused. Aspirin was started at a low dose (4 mg/kg/day). 2D echocardiography was done on day 14, which was within normal limits with no evidence of coronary artery abnormalities. The child gradually improved and became afebrile, experienced a reduction in desquamation, and became playful and active.

## Discussion

KD is typically diagnosed in pediatric patients younger than five, with a male-to-female ratio of approximately 1.5 to 1 [1]. More than five days of fever with at least four other clinical characteristics characterize classic KD: polymorphous skin rash, changes in the limbs, unilateral painless cervical lymphadenopathy, bilateral nonexudative conjunctival injection, and erythema of the lips and oral mucosa [1,3]. Incomplete KD has been used to describe patients with incomplete disease presentation, with or without coronary complications [4]. The immune response in KD is a cascade of systemic inflammation in multiple organs and systems affecting the medium-sized arteries during the acute febrile phase with clinical manifestations, which may include gastroenteritis, aseptic meningitis, interstitial pneumonitis, lymphadenopathy, pyuria, acute pancreatitis, and acute hepatitis [4,9].

Muscular arteries are typically affected by vasculopathy in medium-sized blood vessels. Aneurysms are caused by necrotizing arteritis, vasculitis, and coronary artery damage. Giant saccular aneurysms without intima, medium, or elastica cannot regenerate, but rim rupture or consecutive thrombosis can organize, recanalize, and calcify the residual adventitia [4-9]. Diagnosis of KD is established clinically by clinical findings, and lab criteria may be used to supplement the diagnosis when it cannot be established clinically. To detect coronary aneurysms and other cardiac abnormalities, TTE is the imaging modality of choice.

The underlined incidence of atypical KD may vary from 15% to 36% and is more commonly seen in patients of the younger age group [7,8]. Idris et al. described a four-month-old with high-grade fever with atypical KD [10]. Raut et al. underlined a case of incomplete KD in an infant induced by COVID-19, who presented with high-grade fever and rash [11]. Thadchanamoorthy et al. reported a case of atypical KD in a young child presented with macroscopic hematuria [12]. The American Heart Association (AHA) diagnostic criteria for incomplete KD include unexplained fever for five days and 2/3 acute phase clinical symptoms in infants under six months old. AHA guidelines for incomplete Kawasaki illness include six additional laboratory and echocardiographic criteria [2]. To diagnose atypical KD, a patient should have more than three laboratory abnormalities, which include anemia for age, sterile pyuria, hemoglobinemia (≤ 3g/dl), leukocytosis (≥ 15000/mm^3^), and elevation of alanine aminotransferase and platelet cell count. Infrequent clinical findings underlined in incomplete KD were lymphadenopathy, extremity changes, hyponatremia, and sterile pyuria [13,14]. The patient reported here had extremity changes and sterile pyuria. Manlhiot et al. found identical rates of coronary vessel anomalies in both patient groups using the exact criteria of incomplete KD, which excludes echocardiography [12]. In patients with atypical KD, the period following the onset of symptoms and diagnosis has been extended, and they are considered in the minor category for receiving IVIG [15,16]. Similarly, in this case, a long period was seen between the onset and diagnosis of incomplete KD (~12 days) and was managed with aspirin only. In this case, our patient was diagnosed with incomplete KD because of the lack of the classic diagnostic criteria of KD and the presence of fewer conventional manifestations. After commencing low-dose aspirin, the patient's condition improved.

## Conclusions

Incomplete KD is characterized by the presence of fever for more than five days and the presence of fewer than four signs of mucocutaneous inflammation. It is primarily seen in infants under one year of age and may present with complications such as shock and renal failure. Incomplete KD should be considered in a case of febrile illness that does not adequately meet the criteria and is supplemented by laboratory findings per AHA's guidelines.
